# Evaluation of the agreement of horizontal and vertical linear measurements obtained from digital models, printed models and direct measurements

**DOI:** 10.1590/2177-6709.29.5.e242460.oar

**Published:** 2024-10-07

**Authors:** Sergio Luiz MOTA-JÚNIOR, Ana Clara Titoneli ABREU, Brenda Vitória Monteiro dos SANTOS, Ana Laura Lassance MARANGON, Marcio José da Silva CAMPOS, Robert Willer Farinazzo VITRAL

**Affiliations:** 1 Universidade Federal de Juiz de Fora, Faculdade de Odontologia, Departamento de Ortodontia (Juiz de Fora/MG, Brazil).; 2 Universidade Federal de Juiz de Fora, Faculdade de Odontologia (Juiz de Fora/MG, Brazil).

**Keywords:** Three-dimensional printing, Digital technology, Orthodontics, Impressão tridimensional, Tecnologia digital, Ortodontia

## Abstract

**Introduction:**

The use of arch models is essential in diagnosis and planning in orthodontics. The demand for digital and printed models has increased among professionals.

**Objective:**

The aim of the study was to assess the agreement of horizontal and vertical linear measurements obtained from digital models, printed models, and direct measurements.

**Materials and Methods:**

Intraoral scans of 30 individuals were obtained. Digital measurements were performed using the STL files. From printed models, the measurements were done using a digital caliper, and the real measurements were done directly to the mouth of respective patients.

**Results:**

The one-sample *t* test showed no discrepancy between the paired sets of measurements, with the value of 0 (*p*>0.05). The evaluation of the measurements was done using Bland-Altman analysis in pairs. The three methods showed agreement in horizontal and vertical measurements. Linear regression analyses showed no proportional bias in the data (*p*>0.05).

**Conclusion:**

The horizontal and vertical measurements evaluated showed agreement when measured on digital models, printed models and directly in the individuals’ mouths.

## RESUMO

**Introdução:**

O uso de modelos ortodônticos é essencial no diagnóstico e planejamento em Ortodontia. A demanda por modelos digitais e impressos aumentou entre os profissionais.

**Objetivo:**

O objetivo desse estudo foi avaliar a concordância das medições lineares horizontais e verticais feitas em modelos digitais, modelos impressos e medições diretas.

**Material e Métodos:**

Foram obtidos escaneamentos intrabucais de trinta indivíduos. As medições digitais foram realizadas usando os arquivos STL; as medições nos modelos impressos foram feitas usando um paquímetro digital, e as medições reais foram feitas diretamente na boca dos respectivos pacientes.

**Resultados:**

O teste *t* de uma amostra mostrou que não houve discrepância entre os conjuntos pareados de medições, com valor de 0 (*p*>0,05). A avaliação das medições foi feita com análise de Bland-Altman em pares. Os três métodos mostraram concordância nas medições horizontais e verticais. As análises de regressão linear mostraram que não houve viés proporcional nos dados (*p*>0,05).

**Conclusão:**

As medições horizontais e verticais avaliadas mostraram concordância quando feitas em modelos digitais, modelos impressos e diretamente na boca dos pacientes.

## INTRODUCTION

The use of dental models is an essential step in obtaining the diagnosis and determining the orthodontic treatment plan, serving as a means of communication between professionals and patients.^1^ In this way, it allows the orthodontist to understand the problems to be corrected, the specific challenges of each treatment, and also the possible orthodontic mechanics to be employed during the correction of malocclusion.^2^ ^,^ ^3^

Through the advances in biomaterials, dental plaster models, robust and dimensionally accurate, are considered the gold standard in orthodontics.^4^ ^,^ ^5^ For orthodontics, the most important feature of a digital model system lies in its accuracy and diagnostic reliability.^6^

Although the consensus is that measurements with digital models compare well with those derived from plaster models,^4^ ^,^ ^7^ several studies investigating measurements such as available space, irregularity index, and Bolton analysis have indicated that the average differences between plaster and digital models can exceed 1.5 mm.^8^ ^,^ ^9^ Such a significant difference may not be clinically acceptable. However, there is also conflicting evidence in the literature supporting the validity of digital models for the mentioned measurements.^8^ ^,^ ^10^ ^-^ ^12^

The aim of the present study was to assess the agreement of horizontal and vertical linear measurements obtained from digital models, printed models, and direct measurements in the respective patients. The null hypothesis was that there would be no difference between measurements obtained from digital models, printed models and those obtained directly from patients.

## MATERIAL AND METHODS

This transversal study was approved by the Ethics in Research Committee of Juiz de Fora Federal University (Brazil), with the registration number 49271421.6.0000.5147.

A power analysis was done based on a similar previous study.^13^ A total of 30 individuals were required for this study to obtain an 85% probability of generating 95% confidence intervals with margins of error less than 0.25 mm for horizontal measurements, and 0.1 mm for vertical measurements.

**Figure 1:**
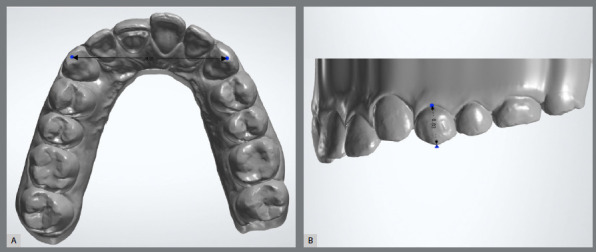
Measurements on the digital model: A) horizontal measurements, B) vertical measurement.

The study sample comprised 30 individuals. The inclusion criteria were: agreeing to voluntarily participate in the sample of the present study, having all permanent teeth erupted, except for the third molars, and being over 18 years old. The exclusion criteria were: presenting wear on the buccal, incisal, or cusp tip surfaces that would hinder the measurement of these teeth. All participants signed an informed consent form. The maxillary and mandibular arches from each individual were scanned using the Trios 3 scanner (3Shape Inc, Copenhagen, Denmark). Once the scans were completed, the raw images were converted to stereolithography (STL) files. These files were used with the commercial software 3Shape 3D Viewer (version 1.3.2, 3Shape Inc, Copenhagen, Denmark) for the digital measurements. The measurements were carried out on digital models with the software’s built-in ruler tool “Measure distance” (Fig. 1).

**Figure 2:**
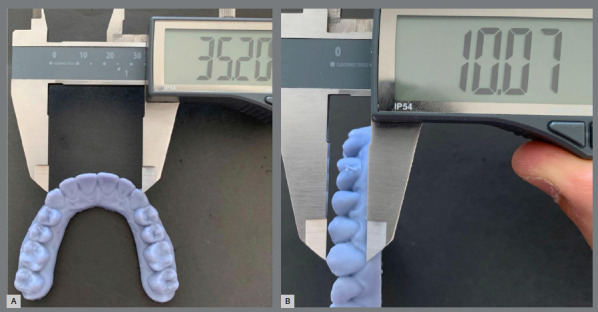
Measurements on the printed model: A) horizontal measurement. B) vertical measurement.

STL files were printed using the 3D Photon S (Anycubic^®^, Shenzhen, China), with the 3D Printing UV Sensitive Resin Grey 405nm (Anycubic^®^, Shenzhen, China). The models were washed with 99.9% isopropyl alcohol, and exposed to ultraviolet light for final curing for 10 minutes in the Wash & Cure 2.0 (Anycubic^®^, Shenzhen, China), according to orientations by the resin manufacturer. A six-inch digital caliper with 0.01 mm precision (IP54; Clockwise Tools, Valencia, USA) was used to make measurements on the printed models (Fig 2), and a bow divider (ICE^®^, Cajamar, Brazil) was used to make the measurements directly on the tooth landmarks (Table 1). All measurements were carried out by the same operator (SLMJ). The digital caliper was utilized because it is a commonly employed instrument in orthodontic practice for measuring dental casts. However, its use is not suitable for direct intraoral measurements, due to physical limitations. Consequently, a bow divider was used for direct intraoral measurements, despite an additional step was required to measure the opening of this device. The operator was trained in a pilot sample with ten cases for each measurement method, until ICC was above 0.9.

**Table 1:** Measurements used and their respective descriptions.

Plane	Measurement	Description
Horizontal	Inter maxillary first molars	Distance between the mesio-buccal cusp tips of the maxillary first molars
	Inter maxillary second molars	Distance between the mesio-buccal cusp tips of the maxillary second molars
	Inter maxillary first premolars	Distance between the vestibular cusp tips of the maxillary first premolars
	Inter maxillary second premolars	Distance between the vestibular cusp tips of the maxillary second premolars
	Inter maxillary canines	Distance between the cusp tip of the maxillary canines
	Inter mandibular first molars	Distance between the mesio-buccal cusp tips of the mandibular first molars
	Inter mandibular second molars	Distance between the mesio-buccal cusp tips of the mandibular second molars
	Inter mandibular first premolars	Distance between the vestibular cusp tips of the mandibular first premolars
	Inter mandibular second premolars	Distance between the vestibular cusp tips of the mandibular second premolars
	Inter mandibular canines	Distance between the cusp tip of the mandibular canines
Vertical	Height maxillary left central incisor	Height along the long axis of the crown of the maxillary left central incisor
	Height maxillary right central incisor	Height along the long axis of the crown of the maxillary right central incisor
	Height maxillary left canine	Height along the long axis of the crown of the maxillary left canine
	Height maxillary right canine	Height along the long axis of the crown of the maxillary right canine
	Height maxillary left first molar	Height along the long axis of the crown of the maxillary left first molar
	Height maxillary right first molar	Height along the long axis of the crown of the maxillary right first molar
	Height mandibular left central incisor	Height along the long axis of the crown of the mandibular left central incisor
	Height mandibular right central incisor	Height along the long axis of the crown of the mandibular right central incisor
	Height mandibular left canine	Height along the long axis of the crown of the mandibular left canine
	Height mandibular right canine	Height along the long axis of the crown of the mandibular right canine
	Height mandibular left first molar	Height along the long axis of the crown of the mandibular left first molar
	Height mandibular right first molar	Height along the long axis of the crown of the mandibular right first molar

The measurements were conducted at three different stages. First, the operator performed the direct measurements from all individuals in the sample. In the second stage, measurements were performed on the printed models, and finally, digital measurements were performed. No measurements with different types of instruments were performed on the same day. Measurements on the printed and digital models were done without any visible identification of the individuals. Another researcher ACT was responsible for identifying the models (with a tape at the base of the printed model with an identification number, and this number was used to name the STL file in the digital model) and tabulating the data, to ensure the blinding of the operator who conducted the measurements. Direct measurements were obtained based on the alphabetical order of the individuals’ first name. Randomization for the order of measurements of the printed and digital models was done using the website *www.randomizer.org*.

SPSS software (version 24; IBM, Armonk, USA) was used for statistical analysis. All measurements were repeated after 30 days, to evaluate the intraclass correlation coefficient (ICC).

A one-sample *t* test was used to assess whether there was any discrepancy between the paired sets of measurements, with the test value set at 0. No statistically significant differences were observed across any of these comparisons. As a result, the three measurements obtained from each individual were combined to represent the virtual model, printed model, and direct measurements, for subsequent analysis. Bland-Altman^14^ analysis was conducted using Microsoft Excel (version 16 for MacOS; Redmond, USA) and SPSS, with Bland-Altman plots generated for the paired comparisons of the three methods. This analysis served to visually illustrate the agreement for both horizontal and vertical values among the digital model measurements, printed model measurements, and direct measurements. Additionally, linear regression analyses were performed to evaluate the presence of any proportional bias in the data. A significance level of *p*<0.05 was adopted for all tests.

## RESULTS

**Figure 3:**
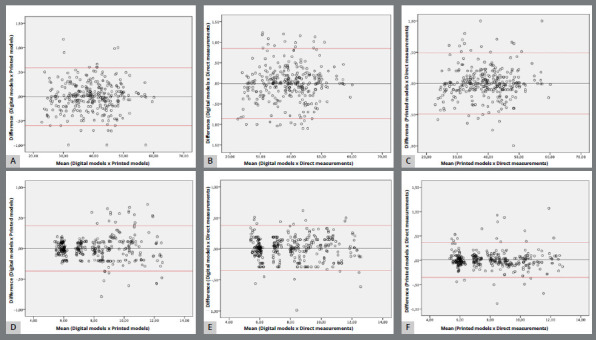
Bland-Altman plot for the comparison of Digital models, Printed models, and Direct measurements for horizontal (**A, B, C**) and vertical (**D, E,** F) measurements.

Repeatability for each set of paired measurements demonstrated excellent results, with intraclass correlation coefficients exceeding 0.99. Table 2 presents the mean biases, standard deviations, confidence intervals, and *p*-values for the paired comparisons of methods. The one-sample *t* test revealed no significant differences among any of the paired methods, suggesting a considerable level of agreement among the tested methods. Figure 3 shows the Bland-Altman plots illustrating the method comparisons. Overall, the Bland-Altman analysis indicated no fixed bias among the three evaluated methods when compared pairwise, and random errors were identified in all comparisons. The mean biases of printed model measurements and directly measurements, in comparison to digital measurements, were -0.08 mm and -0.01 mm, respectively. The lowest minimum mean bias was observed for the comparison between printed model measurements and direct measurements (0.00 mm, 95% confidence interval and agreement limits of -0.03 and 0.03).

**Table 2:** Mean biases, standard deviations (SD), confidence intervals (CI), and *p*-values for method comparisons.

Methods	Mean bias	SD	95% CI	p
Digital x Printed	-0.08	0.30	-0.04 - 0.03	0.633
Digital x Direct	-0.01	0.44	-0.06 - 0.04	0.768
Printed x Direct	0.00	0.25	-0.03 - 0.03	0.945

In each graph in Figure 3, the central line value represents the mean difference between the methods evaluated. Each point on the graph indicates the difference between the value obtained by one method and the value obtained by the other method (compared pairwise in each graph). Across all graphs, the central line is close to 0.00, indicating that the methods were concordant. The two extreme lines (one upper and one lower) represent the 95% confidence interval. Values within this confidence interval demonstrate substantial agreement between the evaluated methods. Although there are points outside the confidence interval (above the upper line or below the lower line), these points are random, since there are 10 horizontal measurements and 12 vertical measurements per individual.

The outcomes of the linear regression analyses are outlined in Table 3. None of the comparisons demonstrated statistical significance for the *t* scores, suggesting the absence of proportional bias in any comparison. Consequently, the null hypothesis was accepted, indicating the absence of any discernible trend for mean differences above or below the mean bias level depicted in the Bland-Altman scattergram plots.

**Table 3:** Summary of the linear regression analysis.

Plane	Measurement	t	P
Horizontal	Digital x Printed	0.42	0.676
	Digital x Direct	1.14	0.257
	Printed x Direct	0.98	0.328
Vertical	Digital x Printed	0.76	0.449
	Digital x Direct	-0.35	0.729
	Printed x Direct	-1.15	0.251

## DISCUSSION

The reliability and accuracy of measurement methods used in orthodontics play a crucial role in determining accurate diagnoses and formulating effective treatment plans.^2^ In this study, the results obtained from digital models, printed models, and direct measurements in the patient’s mouth were compared to evaluate agreement between measurements. To our knowledge, this is the first report to evaluate digital, printed and direct measurements. The Bland-Altman^14^ method was chosen because it is indicated for evaluating the agreement between different measurement methods. In this way, comparisons were made in pairs, and it was observed that the methods were in agreement with each other, showing that it is reliable to obtain vertical and horizontal measurements in digital and printed models for use in diagnosis and planning in orthodontics. The findings revealed a high agreement among the measurement methods, with all comparisons showing statistically non-significant differences. This indicates that all methods were equally reliable in obtaining measurements.

Digital models are as reliable as traditional plaster models, exhibiting high precision, reliability, and reproducibility.^15^ ^-^ ^17^ The identification of the reference points, rather than the measuring device or software, appears to be the primary limitation when using digital models.^18^ Additionally, with their advantages in terms of cost, time, and space required, digital models can be considered the new gold standard in current practice.^18^ According to the present study, digital models presented the same measurement accuracy as printed models and measurements obtained directly in the mouth, confirming the advantage of being a reliable method for collecting measurements used in orthodontics, and can be reliable substitutes for plaster models.

Furthermore, the validity of the measurement methods was confirmed by the consistency of results obtained between digital models, printed models, and direct measurements in the patient’s mouth. The statistical comparability among these methods suggests that they are all equally valid for evaluating the orthodontic variables investigated in this study. These results are encouraging, as they indicate that orthodontists can rely on a variety of measurement methods to inform their diagnoses and treatment plans without compromising accuracy or reliability.

Although impression materials and plaster models are very accepted, they are prone to deformation,^19^ and can be considered a less comfortable method for patients than digital methods using intraoral scanning.^20^ Thus, we used a bow divider to obtain measurements directly in the mouth, instead of using a plaster model, and it was possible to evaluate the three obtained methods in pairs. Polyjet printers can be considered more accurate than digital light processing (DPL),^13^ ^,^ ^21^ as they are capable of printing 16µm details, while DLP printers print 50µm;^22^ however, for use in orthodontics, models printed with DLP and polyjet are appropriate to be used.^13^ ^,^ ^22^ ^,^ ^23^ The DLP was chosen for the study because this is our clinical use printer.

From a clinical standpoint, the reliability and statistical equivalence among measurement methods have significant implications in orthodontic practice. Orthodontists can select the most suitable measurement method based on practical considerations and individual preferences, without compromising the quality of results. This allows for a personalized approach to treatment planning, taking into account the specific needs and circumstances of each patient.

However, it is important to acknowledge some limitations of this study, such as the selection of specific variables for evaluation. It is suggested that future research further explores the applicability of measurement methods in different clinical scenarios, like tooth wear, restoration and gingival recession, and investigates other orthodontic variables not addressed in this study. With a more comprehensive understanding of the reliability and validity of available measurement methods, orthodontists can further improve the accuracy of their diagnoses and treatment plans, providing more effective and personalized orthodontic care.

## CONCLUSION

The horizontal and vertical measurements evaluated showed agreement when measured on digital models, printed models and directly in the individuals’ mouths.
